# Applying taper function models for black locust plantations in Greek post-mining areas

**DOI:** 10.1038/s41598-024-63048-1

**Published:** 2024-06-12

**Authors:** Florian Wilms, Ferréol Berendt, Karol Bronisz, Ulyana Bashutska, Mariangela Fotelli, Kalliopi Radoglou, Gavriil Spyroglou

**Affiliations:** 1 Forest Research Institute, Hellenic Agricultural Organization DIMITRA, 57006 Thessaloniki, Greece; 2 Department of Forest Utilization and Timber Markets, Eberswalde University for Sustainable Development, 16225 Eberswalde, Germany; 3 Institute of Forest Sciences, Warsaw University of Life Sciences-SGGW, 02-776 Warsaw, Poland; 4 Department of Ecology, Ukrainian National Forestry University, Lviv, 79057 Ukraine; 5 Department of Forestry and Management of the Environment and Natural Resources, Democritus University of Thrace, 68200 Orestiada, Greece

**Keywords:** Forest landscape restoration, Stem taper, Tree volume, Fixed- and mixed-effects modelling, Random coefficients, *Robinia pseudoacacia* L., Forestry, Ecological modelling

## Abstract

A key process in forest management planning is the estimation of tree volume and, more specifically, merchantable volume. The ability to predict the cumulative stem volume relative to any upper stem diameter on standing trees or stands is essential for forest inventories and the management of forest resources. In the 1980s, the Hellenic Public Power Corporation (HPPC) started the rehabilitation of lignite post-mining areas in Greece by planting mainly black locust (*Robinia pseudoacacia,* L.). Today, these plantations occupy an area of approximately 2570 ha, but the stem volume has not yet been estimated. Therefore, we aimed to estimate the over- and under-bark stem volume using taper function models for 30 destructively sampled trees. Of the nineteen calibrated fixed-effects models, Kozak’s (2004) equation performed best for both the over-bark and under-bark datasets, followed by Lee’s (2003) and Muhairwe’s (1999) equations. Two fixed effect models were compared with fitted coefficients from Poland and the United States confirming that the local model fits were better suited, as the foreign model coefficients caused an increase in root mean square error (RMSE) for stem diameter predictions of 13% and 218%, respectively. The addition of random effects on a single-stem basis for two coefficients of Kozak’s (2004) equation improved the model fit significantly at 86% of the over-bark fixed effect RMSE and 69% for the under-bark model. Integrated taper functions were found to slightly outperform three volume equations for predictions of single stem volume over and under bark. Ultimately it was shown that these models can be used to precisely predict stem diameters and total stem volume for the population average as well as for specific trees of the black locust plantations in the study area.

## Introduction

Black locust (*Robinia pseudoacacia*, L.) is a tree species native to North America. The medium-sized, deciduous, thorny, fast-growing species grows on a wide range of soil types and is one of the most important and widespread broadleaved alien tree species in Europe^1^. The ability of black locust to perform well in various habitats, especially during early succession, is noted in the review of Cierjacks and others^2^. The authors refer to the documentation of black locust invasion on abandoned gravel-sand pits and landfills, urban brownfield sites, secondary forests on farmland, coppice forests and pastures, along disturbed roadsides and on burned sites. Black locust was first introduced to Europe at the beginning of the seventeenth century^3^ and is now the second most often planted broadleaved tree species worldwide, after eucalyptus^4^. The resistance of black locust to heat and drought, its salt tolerance, and its symbiosis with nitrogen-fixing bacteria are particularly important under extreme environmental conditions and therefore for the rehabilitation of degraded areas^5–7^. Because of these ecological and biological properties, black locust is often planted in the steppe zone of Ukraine to create forest plantations, rehabilitate forests and reclaim degraded soils^8–11^. Black locust is widely used to obtain above-ground biomass in short rotation coppices^12–16^. Moreover, it is a suitable tree species for the restoration of sites that have been significantly degraded through anthropogenic activities, such as former open-cast mining areas^17^. However, there is some concern about the future management of black locust in Europe due to its possible ecological disadvantages related to invasiveness, threats to biodiversity and induced changes in microclimate and soil traits^18–21^.

In Greece, the National Forest Service introduced and planted black locust to stabilize torrents on mountains and prevent soil erosion near rivers, roads and railway banks. It has also been used as fodder in silvopastures^22^ and as an alternative plantation crop for privately owned marginal agricultural lands, in line with the 2080/92 and 1257/99 European Union (EU) Regulations^23^. In the 1980s, the Hellenic Public Power Corporation (HPPC) started to rehabilitate open-cast coal mining fields in the Lignite Center of Northwestern Greece by planting mainly black locust, both alone and in mixture with other species. The establishment of plantations is still ongoing, but already with a planted area of 2570 ha, the HPPC is the largest private forest plantation owner in Greece, owning 26% of the country’s black locust plantation area^24^. To date, there is no inventory or management plan for those plantations.

One of the key processes in forest management planning is the estimation of tree volume and, more specifically, merchantable volume^25^. There is no accurate and direct method for calculating tree volume due to the unique and complex shape of the stem. Water displacement methods require destructively sampled trees and are both time consuming and expensive. Therefore, tree volume estimation is usually based on empirical—heuristic volume models. Although a tree stem cannot be completely described in mathematical terms, it is convenient to assume that segments of a tree bole approximate various geometric solids. The lower portion of the stem is generally assumed to be a neiloid frustum, the middle portion a paraboloid frustum and the upper portion a cone^26,27^. The form factor approach is widespread in forest practice and is used to calculate volume^28^. A limitation of this method is that while it can result in accurate estimates of whole stem volume, it is not useful for estimating tree section or log volumes. This problem can be overcome by describing the stem profile through the development of taper curves and equations. There are two common methodologies for obtaining taper and volume models^29,30^. One is to develop volume ratio equations that predict merchantable volume as a percentage of total tree volume^31^; the other is to define an equation describing the stem taper.

Taper equations are flexible models that can provide estimates of (1) total stem volume, (2) merchantable volume to any top diameter and from any stump height diameter, (3) log volumes of any length at any height from the ground and (4) biomass^32^. Moreover, taper equations may be used to produce assortment tables based on log lengths or specific diameter thresholds^25,33^. Closely related volume models can only estimate the total or merchantable volume of a tree. Tree taper is important for making accurate estimates of standing timber, especially in terms of product potential. Taper equations can be useful in predicting the volume of an individual tree or a stand of trees^34,35^. Many empirical models have been used to describe tree profiles. Ormerod^36^ cited Höjer’s^37^ logarithmic equation as the first attempt to model tree taper. Research has shown that stem profile could not be described with simple equations. The idea was that the new taper equations should produce the whole stem volume estimate as existing volume equations. Goulding and Murray^38^ provided the so-called compatible taper equations, but these equations exhibited significant bias in certain regions of the stem. Grosenbauch^39^ noted that tree profile is best modeled in sections and that the necessary conditions are that the sub-models produce identical diameters at joint points and that diameters decrease monotonically from the base of tree to the top. Max and Burkhart^40^ used their segmented polynomial model and demonstrated the superiority of three sub-models over two. Thomas and Parresol^41^ applied flexible trigonometric taper equations for both coniferous and broadleaved tree forms.

Many forms and types of stem profile models have been published^42^ and evaluated for accuracy and precision^43–45^. Martin^45^ evaluated five profile models for predicting diameter, height, and volume and found that no single model performed best for all applications. He reported that the Max and Burkhart^40^ model was best overall. Cao and others^44^ evaluated six profile models for predicting diameter and ranked Max and Burkhart’s segmented-profile polynomial model first and Cao’s segmented-profile model second. Cao’s segmented-profile model was ranked first for estimating volumes to various top diameters. A polynomial ratio model was ranked second, Burkhart’s^31^ ratio model was third, and Max and Burkhart’s segmented-profile model was fourth. The Alberta Forest Service^43^ evaluated 15 stem-profile models and judged Max and Burkhart’s model best because of its accuracy and ease of computation.

While the number of taper equations now available has grown significantly, they can generally be classified into two categories: parametric and non-parametric equations^46^. Parametric approaches include polynomial, trigonometric and variable-exponent equations. Segmented polynomial equations, e.g. Max and Burkhart 1976^40^, contain conditional clauses with different parts of the function applying to the taper depending on relative height along the stem. Elaborate functions with a large number of independent variables and coefficients to be estimated, especially variable-exponent equations^33,47^ are often found to perform better than their less complex counterparts^48^.

The empirical data intended to create taper models are characterized by a hierarchical structure containing information about individual diameters, trees or sites. One possibility of modeling these data in the case of parametric equations is the mixed-effects modelling approach^49–51^. The fixed component defines the level for a typical basic group (e.g., trees or sites), whereas the random part describes the difference between each group and a typical value. This approach allows both the capture of all dependencies occurring in the analyzed data and the assessment of variability occurring at various levels, as well as the use of the obtained information in the prediction of the modeled parameter (usually the diameter along the tree^48^). On the other hand, machine-learning methods, artificial neural networks especially, have been explored as viable non-parametric approaches to modelling tree taper^52–54^. Calibrated taper models may in turn be applied to predict the timber assortment to be harvested from each stem.

Because there are no models available for calculating the wood volume of black locust stands and no taper functions to estimate the merchantable volume at any stem diameter along the trunk for Greek conditions, the aims of this study were to (i) fit taper equations for black locust from the restored area of the Lignite Center of Northwestern Greece, (ii) compare the taper equations with other published taper models for planted black locust in restored coal mines, and (iii) fit volume equations for tree volume using the best performing taper models.

## Materials and methods

### Site description

**Figure 1:**
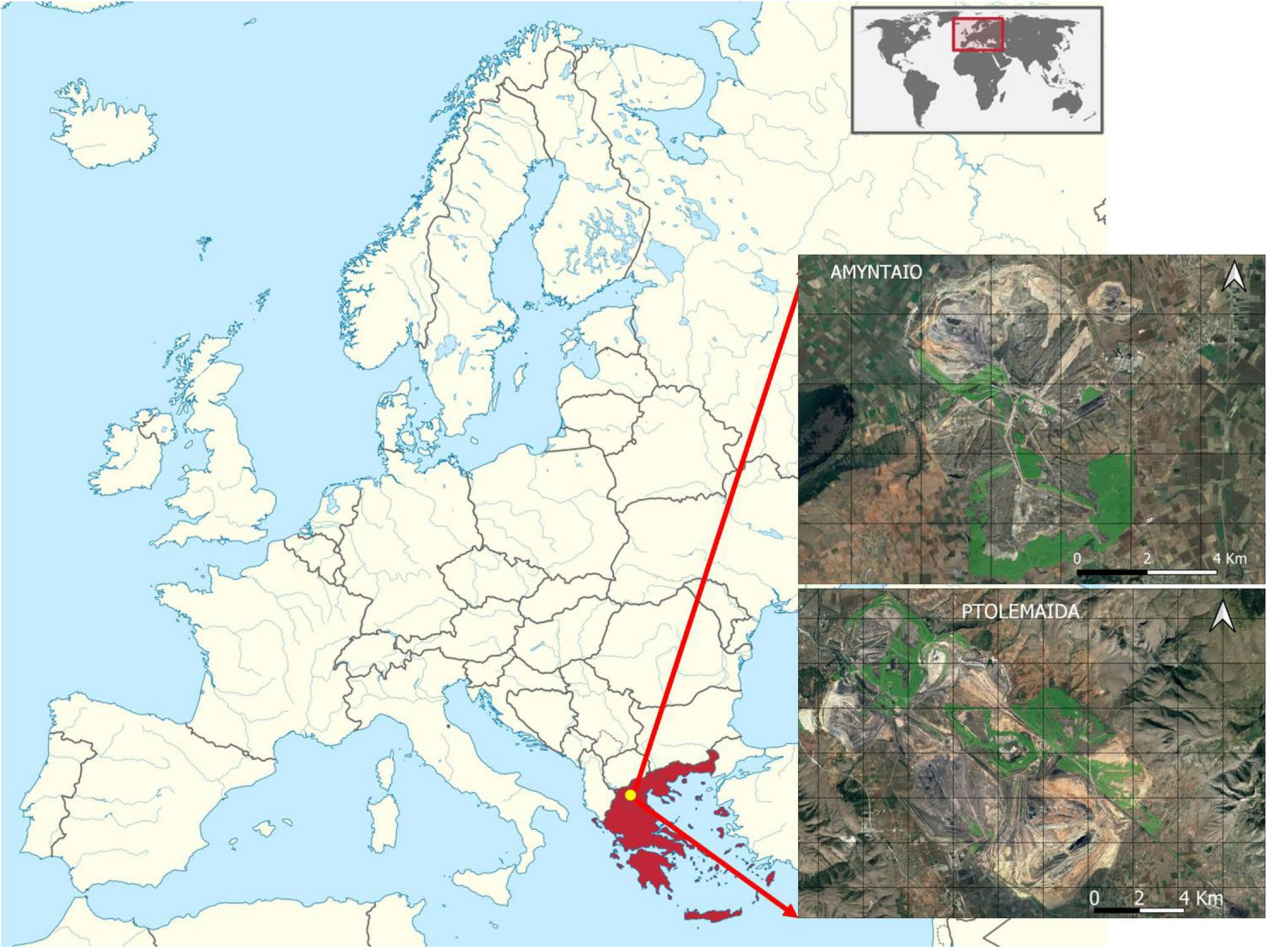
Map of the Amyntaio and Ptolemaida open-cast mine sites. The green colored polygons represent the restored areas planted with black locust.

In this study, we inventoried the black locust restoration plantations on the former open-cast mining areas of the lignite center of Northwestern Greece (Fig. 1). The plantations are located near Amyntaio (40.56° to 40.61° N and 21.62° to 21.69° E) and Ptolemaida (40.39° to 40.51° N and 21.7° to 21.89° E). Black locust covers more than 95% of the planted area, followed by weaver’s broom (*Spartium junceum* L) and Arizona cypress (*Cupressus arizonica* Greene), covering 2.45% and 1.44%, respectively. Other planted species include oaks, maples, pines and various deciduous broadleaves but these make up only very small percentages of the total planted area. The plantations are established on open-cast mining deposits with varying topography (moderate to steep slopes, plains and terraces) and altitudes ranging from 530 to 950 m. a.s.l. The landscape is fragmented by different land uses, which, in addition to forest plantations, also include grasslands, agricultural lands and bare lands used for photovoltaic parks and recycling facilities. The air temperature of the region ranges from 6.1 to 17.4 °C with a mean annual temperature of 12.2 °C. The total annual precipitation is 664 mm (mean values over the last 50 years).

### Sampling and measurements

Sampling took place in August–September 2019 and July–September 2020. In total, 30 trees representing the locally present diameter range (3–22 cm) were destructively sampled. Each tree was then cut into 6 sections using a chainsaw or branch cutter, depending on diameter. The sections are located at (1) stump height = 0.3 m, (2) breast height = 1.3 m, (3) mid-bole height, which was defined as the middle point between the ground and crown base height, (4) live crown base height, (5) lower third of the crown, and (6) upper third of the crown. The crown base height was defined as the lowest knot of a live branch.

The stem and branch section discs were sanded and subsequently scanned with an Expression 11000XL scanner (Seiko Epson, Japan). Tree ring increment and section diameters were digitally recorded using WinDENDRO 6.0.4 software^55^ and analyzed via the associated XLStem add-in^56^ for Microsoft Excel^57^.

On each disc, increment paths were identified in four perpendicular directions. For the sections between ground and crown base (sections 1–4), one disc was identified and digitally analyzed. For the crown sections (sections 5 and 6), every branch dissecting the respective imaginary line was cut and a disc was sampled. The three discs with the largest diameters per crown section were subsequently used for further analysis in WinDENDRO^55^. In cases where discs were damaged due to stripped bark or being rotten, the digital measurements were conducted on the next intact disc. The reference stem volume [m^3^] was calculated via Smalian’s equation by the XLStem analysis output.

### Taper equations for fixed-effect models

Since the nature of taper datasets violates the assumption of homogenous variance, modelling was not conducted via the basic nonlinear least squares (*nls*) approach. Instead, using the generalized nonlinear least squares function *gnls* of the nlme package^58^, a power type function was applied to model residual variance, as reported by Bronisz and Zasada^49^. Additionally, an autoregressive structure of order 1 was applied on a stem basis.

Some taper equations use only a single explanatory variable such as the segmented model^40^ or the trigonometric model^41^, while others use up to four explanatory variables^59,60^. The selection criteria for taper equations were to include this spectrum of simple to complex functions, while also focusing on those that were shown in the literature to perform well.

In order to predict the same dependent variable ‘section diameter squared’, all functions were modified according to Bruce et al.^61^ and de-Miguel et al.^48^.

Nomenclature:

$$D$$
section diameter [cm], over or under bark
$$DBH$$
diameter o.b. [cm] at breast height
$$H$$
total tree height [m]
$$HD$$
section height [m]
$$RH$$
$\frac{HD}{H}$ relative height: section height [m] / total tree height [m]
$$\beta 1 \ldots \beta 10$$
fixed-effect parameters to be calibrated

Taper equations:

Bi and Long^62^

1 $$D^{2} \sim \left\{ {\left[ {\frac{{\ln \left( {\sin \left( {\frac{\pi }{2}*RH} \right)} \right)}}{{\ln \left( {\sin \left( {\frac{\pi }{2}*t} \right)} \right)}}} \right]^{{\beta 1 + \beta 2*\sin \left( {\frac{\pi }{2}*RH} \right) + \beta 3*\cos \left( {\frac{3\pi }{2}*RH} \right) + \beta 4*\frac{{\sin \left( {\frac{\pi }{2}*RH} \right)}}{RH} + \beta 5*D + \beta 6*RH*\sqrt D + \beta 7*RH*\sqrt H }} *DBH} \right\}^{2}$$

where $t=\frac{1.3m}{H}$, all other variables are described above.

Biging^63^

2 $$D^{2} \sim \left\{ {\left[ { \beta 1 + \beta 2*\ln \left( {1 - RH^{\frac{1}{3}} } \right)*\left( {1 - e^{{\left( { - \frac{\beta 1}{{\beta 2}}} \right)}} } \right)} \right]*DBH} \right\}^{2}$$

Bruce et al.^61^

3 $$\begin{gathered} D^{2} \sim \left\{ {\left[ {\beta 1*k^{0.1} + \beta 2*\left[ {k^{1.5} - k^{3} } \right]*DBH*0.01 + \beta 3*\left[ {k^{1.5} - k^{3} } \right]*H*0.001 + \beta 4*\left[ {k^{1.5} - k^{32} } \right]*HD*0.0001} \right.} \right. \hfill \\ \left. {\left. {\quad \quad + \beta 5*\left[ {k^{1.5} - k^{32} } \right]*H^{0.5} *0.001 + \beta 6*\left[ {k^{1.5} - k^{40} } \right]*H^{2} *0.00001} \right]*DBH} \right\}^{2} \hfill \\ \end{gathered}$$

Cao^44^ as found in Parresol et al.^64^

4 $$D^{2} { }\sim { }\left[ {\sqrt {\begin{array}{*{20}c} {2*z + \beta 1*\left( {3{*}z^{2} - 2{*}z} \right) + \beta 2*\left( {z - b4} \right)^{2} *\left( {z \ge b5} \right) + } \\ {\beta 3*\left( {z - \beta 4} \right)^{2} *\left( {z \ge \beta 5} \right)} \\ \end{array} } {*}DBH} \right]^{2}$$

where $z = \frac{H - HD}{H}$, all other variables are described above

Demaerschalk^65^

5 $$D^{2} \sim \left[ {\beta 1*DBH^{\beta 2} *\left( {H - HD} \right)^{\beta 3} *H^{\beta 4} } \right]^{2}$$

Kozak et al.^66^

6 $$D^{2} \sim \left\{ {\left[ {\beta 1*\left( {RH - 1} \right) + \beta 2*\left( {RH^{2} - 1} \right)} \right]*DBH} \right\}^{2}$$

Kozak^59^

7 $${\text{D}}^{2} = \left[ {\beta 1\left( {DBH^{\beta 2} } \right) + \left( {H^{\beta 3} } \right) + \left( {\frac{{1 - \sqrt {RH} }}{1 - \sqrt t }} \right)^{{\beta 4^{0.1} }} + \beta 5\left( {RH^{4} } \right) + \beta 6*\arcsin \left( { 1 - \sqrt {RH} } \right) + \beta 7*\left( {\frac{1}{{e^{{{\raise0.7ex\hbox{${DBH}$} \!\mathord{\left/ {\vphantom {{DBH} H}}\right.\kern-0pt} \!\lower0.7ex\hbox{$H$}}}} }}} \right) + \beta 8 *DBH^{{\left( {\frac{{1 - \sqrt {RH} }}{1 - \sqrt t }} \right)}} } \right]^{2}$$

where $t=\frac{1.3m}{H}$, all other variables are described above

Kozak^33^

8 $$D^{2} \sim \left[ {\beta 1*DBH^{\beta 2} *H^{\beta 3} *C^{{\beta 4*H^{4} + \beta 5*\left( {1 - e^{\frac{DBH}{H}} } \right) + \beta 6*C^{0.1} + \beta 7*\frac{1}{DBH} + \beta 8*H^{Q} + \beta 9*C}} } \right]^{2}$$

where $C = { }\frac{{1 - \left( \frac{HD}{H} \right)^{\frac{1}{3}} }}{{1 - \left( {\frac{1.3}{H}} \right)^{\frac{1}{3}} }}$,$Q = { }1 - \left( \frac{HD}{H} \right)^{\frac{1}{3}}$, all other variables are described above

Laasasenaho^67^ as found in de-Miguel et al.^48^

9 $$D^{2} \sim \left\{ {\left[ {\beta 1*RH + \beta 2*RH^{2} + \beta 3*RH^{8} + \beta 4*RH^{34} } \right]*DBH} \right\}^{2}$$

Lee et al.^68^

10 $$D^{2} \sim \beta 1*DBH^{\beta 2} *\left[ {\left( {1 - RH} \right)^{{\left( {\beta 3*RH^{2} + \beta 4*RH + \beta 5} \right)}} } \right]^{2}$$

Max and Burkhart^40^

11 $$D^{2} \sim \left[ {\beta 1\left( {RH - 1} \right) + \beta 2\left( {RH^{2} - 1} \right) - \beta 3\left( {\alpha 1 - RH } \right)^{2} *I_{\beta 5 - RH} + \beta 4\left( {\alpha 2 - RH} \right)^{2} *I_{\beta 6 - RH} } \right]*DBH^{2}$$

where:$${I}_{\beta 5-RH}$$ = 1, $$\beta 5-RH\ge 0$$; $${I}_{\beta 5-RH}$$ = 0, $$\beta 5-RH<0$$; $${I}_{\beta 6-RH}$$ = 1, $$\beta 6-RH\ge 0$$; $${I}_{\beta 6-RH}$$ = 0, $$\beta 6-RH<0$$

Muhairwe^47^

12 $$D^{2} \sim \beta 1*DBH^{\beta 2} *\left[ {\left( {1 - RH} \right)^{{\beta 3*RH + \beta 4*RH^{2} + \frac{\beta 5}{{RH}} + \beta 6*RH^{3} + \beta 7*DBH + \beta 8*\left( \frac{DBH}{H} \right)}} } \right]^{2}$$

Newberry and Burkhart^69^

13 $$D^{2} \sim \left[ { \beta 1*DBH*\left( {\frac{H - HD}{{H - 1.3}}} \right)^{\beta 2} } \right]^{2}$$

Riemer et al.^70^

14 $$D^{2} \sim \left\{ {\frac{\beta 1*DBH}{{1 - e^{{\beta 3*\left( {1.3 - H} \right)}} }} + \left( {\frac{DBH}{2} - \beta 1*DBH} \right)*\left[ {1 - \frac{1}{{1 - e^{{\beta 2*\left( {1.3 - H} \right)}} }}} \right] + e^{ - \beta 2*H} *\left[ {\frac{{\left( {\frac{DBH}{2} - \beta 1*DBH} \right)*e^{1.3*\beta 2} }}{{1 - e^{{\beta 2*\left( {1.3 - H} \right)}} }}} \right] - e^{\beta 3*H} *\left[ {\frac{{\beta 1*DBH*e^{ - \beta 3*H} }}{{1 - e^{{\beta 3*\left( {1.3 - H} \right)}} }}} \right]} \right\}^{2}$$

Sharma and Oderwald^71^

15 $$D^{2} \sim DBH^{2} *\frac{HD}{{1.3}}^{2 - \beta 1} *\frac{H - HD}{{H - 1.3}}$$

Sharma and Zhang^72^

16 $$D^{2} \sim \left\{ {DBH*\beta 1*\left( {\frac{HD}{{1.3}}} \right)^{{\left[ {2 - \left( {\beta 2 + \beta 3*RH + \beta 4*RH^{2} } \right)*\left( {\frac{H - HD}{{H - 1.3}}} \right)} \right]^{0.5} }} } \right\}^{2}$$

Sharma and Parton^73^

17 $$D^{2} \sim \left\{ {DBH*\beta 1*\left( {\frac{HD}{{1.3}}} \right)^{{\left[ {2 - \left( {\beta 2 + \beta 3*RH + \beta 4*RH^{2} } \right)*\left( {\frac{H - HD}{{H - 1.3}}} \right)} \right]^{0.5} }} } \right\}^{2}$$

Thomas and Parressol^41^

18 $$D^{2} = \left[ {\beta 1\left( {RH - 1} \right) + \beta 2 \sin \left( {2*\pi *RH} \right) + \beta 3 \cot an\left( {\pi *\frac{RH}{2}} \right)} \right]*DBH^{2}$$

Westfall and Scott^60^

19 $$D^{2} = DBH^{2} *\left( {\frac{{\frac{1.3}{H}}}{{\beta_{5} }}} \right)^{{\beta_{10} }} *\left( {\frac{{1 - \frac{HD}{H}}}{{1 - \beta_{5} }}} \right)^{{\left( {\varphi_{{1_{j} }} + \beta_{1j} } \right) + \left[ {\frac{{\varphi_{{2_{j} }} + \beta_{7} }}{{\left( {1 + \frac{HD/H}{{\beta_{8} }}} \right)^{{\beta_{9} }} }}} \right]}} * \left( {\frac{{1 - \frac{HD}{H}}}{{1 - \beta_{6} }}} \right)^{{\beta_{2} *\frac{{\left( {\frac{\frac{HD}{H}}{{\beta_{3} }}} \right)^{{\beta_{4} *\frac{DBH}{H}}} }}{{1 + \left( {\frac{\frac{HD}{H}}{{\beta_{3} }}} \right)^{{\beta_{4} *\frac{DBH}{H}}} }}}}$$

where $\varphi 1_{j} , \varphi 2_{j}$—random effects for tree j.

### Comparison with studies on Black locust taper

The predicted values from the present Greek dataset were compared to two sets of calibrated coefficients of closely related publications: the Western Polish model for Kozak’s 1995 taper equation^59^ (Eq. 7) for 48 black locust trees^49^ and the North-Eastern US taper equation (Eq. 19) calibrated with a tree species group including black locust^60^. Both models were analyzed alongside the fixed-effect models for diameters over bark. The lack of fit compared to the present model based on Greek data was adressed via residual plots and correlation testing with a linear model.

### Mixed-effect taper models

As a result of the fixed-effect (FE) model ranking, the best taper model was selected to be fitted with random effects. Mixed-effect (ME) modelling was conducted using the same application of the power type function to model the residual variance as previously described for FE. Random effects were assigned at the tree grouping-level to improve the individual tree fit along the stem, thus accounting for possible differences in taper behaviour e.g. by age. To include all possible combinations of random effects for every coefficient, a grid search with 1, 2 and 3 random effects was performed. ME model performance was ranked using the same selection of goodness-of-fit metrics as the FE models. In the third step, a likelihood-ratio test was applied to confirm a statistically significant difference of coefficients between the best performing ME model and its FE counterpart. Finally, possible decreases in heteroscedasticity through the addition of random effects to the FE model were assessed via residual analysis. Residuals were plotted against the relative height RH along the stem and section diameter D_ob_ and D_ub_.

### Stem volume equations

The reference stem volume [m^3^] was calculated using Smalian’s equation applied to the measured section diameters. The predicted stem volume for the FE models was derived using numerical integration provided by the *integrate()*-function in R. Three volume equations from the literature, previously tested for black locust as a function of DBH and H,^49^ were used for comparison without refitting their coefficients. In contrast to taper equations being fitted to repeated measurements (i.e., section diameters along the stem), the three applied volume functions (Eqs. 20, 21 and 22) require only total stem volume in the fitting procedure, eliminating the autocorrelation . The argument of simplicity prevails, as the volume equation terms do not include conditional clauses (Eqs. 4, 11) or complex exponents (Eqs. 7, 19).

Smalian’s equation

$$V = \mathop \sum \limits_{i \ldots n - 1} \frac{{g_{i} + g_{i + 1} }}{2}*l + V_{n}$$

where ${g}_{i}$ is the cross-sectional area of the $i$-th section, $l$ is the section length and ${V}_{n}$ is the cone at the tip of the tree.

Formulae for stem volume equations

Sopp and Kolosz^74^

20 $$V_{stem} = 10^{ - 8} {*}DBH^{2} {*}H{*}\left[ {\frac{H}{{\left( {H - 1.3} \right)}}} \right]^{2} {*}\left( {0.6326{*}DBH{*}H + 20.23{*}DBH + 3034} \right)$$

Moshki and Lamersdorf^75^

21 $$V_{stem} = 0.4{*}H{*}DBH^{2}$$

Lockow and Lockow^76^

22 $$V_{stem} = { }4.848577837{ *}10^{ - 5} {*}DBH^{1.946545395} {*}H^{0.96112739}$$

### Statistical analysis

All statistical computations were executed using the R programming language, version 4.0.3^77^. The R-packages used for statistical analysis were *minpackLM*^78^ *and nlme*^58^. Graphs were created using *ggplot2*^79^ A significance level of α = 5% was applied to confirm the statistical significance of model coefficients. Five goodness-of-fit metrics were used to evaluate model performance. While it has been argued that Root Mean Square Error ($RMSE$) (Eq. 23) is a worse assessor of average errors than Mean Absolute Error ($MAE$) (Eq. 24)^80^, it is widely used in performance rankings of taper model calibrations and is included here for comparability with other publications. Mean Bias ($MB$) (Eq. 25) and Mean Percent Bias ($MPB$) (Eq. 26) are both used to quantify a trend in the model’s predicted values. $MPB$ was chosen in addition to $MB$ in order to be able to assess bias relative to the actual value. As taper data naturally covers a wide range of diameters, the same absolute error value is increasingly more influential with decreasing diameter. Lastly, Akaike’s Information Criterion ($AIC$) (Eq. 27)^81^ measures the model fit by likelihood testing. $AIC$ favors parameter parsimonious functions, i.e., the model performance receives a better score for fewer parameters being estimated. For $RMSE$, $MAE$ and $AIC$, the lowest values are desired, whereas for $MB$ and $MPB$, values closer to 0 indicate the best performance.

23 $$RMSE = { }\sqrt {\frac{{\sum \left( {y - \hat{y}} \right)^{2} }}{n} }$$

24 $$MAE = \frac{{\sum \left| {y - \hat{y}} \right|}}{n}$$

25 $$MB = { }\frac{{\sum \left( {y - \hat{y}} \right)}}{n}$$

26 $$MPB = { }100{*}\frac{{\sum \left( {y - \hat{y}} \right)}}{\sum y}$$

27 $$AIC = { }2k - 2*{\text{ln}}\left( {\hat{L}} \right)$$

where $y$ = observed value, $\widehat{y}$ = predicted value, $n$ = sample size, $k$ = number of estimated parameters, $\widehat{L}$ = likelihood of the estimator

The residual variance of the fixed and mixed effect models was modelled using a power function of DBH (Eq. 28), where ${\varepsilon }_{ij}$ is the residual of the individual section diameter squared, $\sigma$ being the shape and $\partial$ being the scale parameter. The function was fitted on an individual tree basis, such that each individual sample tree received its own $\partial$, which is reported as the mean ± standard deviation.

28 $$var\left( {\varepsilon_{ij} } \right) = \sigma^{2} DBH_{ij}^{\partial }$$

A model’s relative ${Rank}_{i}$ (Eq. 28) by a certain goodness-of-fit metric (e.g. $RMSE$), i.e., between the best and worst performing model, was calculated using the ranking method of Poudel and Cao^82^:

29 $$Rank_{i} = 1 + { }\frac{{\left( {n - 1} \right){* }\left( {M_{i} - M_{min} } \right)}}{{M_{max} - M_{min} }}$$

where

$n$—number of compared models.

${M}_{i}$—respective evaluated value of the model

${M}_{min}$, ${M}_{max}$—the lowest (mostly best) and highest (worst) overall values of a metric.

The final model performance was assessed by ranking the mean of each metric performance as $RankAVG$.

### Data overview

The dendrochronological analysis showed that the age of the trees ranged between 4 and 24 years. Tree age was determined via ring count at the stump height (HST) section disc (0.3 m). Mean DBH was 10.91 ± 4.52 cm and mean tree height was 11.79 ± 3.28 m (Table 1). Due to the overall small stem dimensions, the volumes of the observed trees were small with a maximum of 0.24 m^3^. Stem volume also displayed the highest coefficient of variation with almost 100% around the mean.

**Table 1:** Summary statistics for diameter at breast height = DBH, tree height = H, crown base height = HCB and stem volume = V (n = 30).

	Mean	± SD	CV%	Min	Max
$DBH$[cm]	10.91	4.52	41.43	3.20	21.93
$H$[m]	11.79	3.28	27.79	5.95	17.80
$HCB$[m]	6.64	2.57	38.69	1.70	11.90
$V$[m^3^]	0.07	0.06	92.34	0.002	0.238

**Figure 2:**
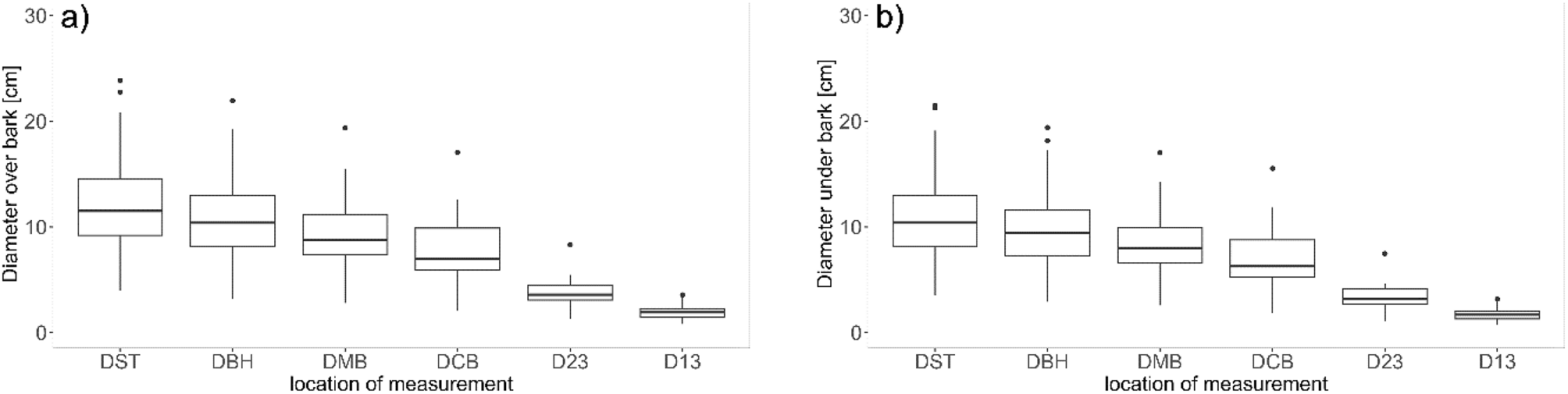
Distribution of over-bark (**a**) and under-bark (**b**) diameters at section heights with DST = diameter at stump height, DBH = diameter at breast height, DMB = diameter at mid-bole, DCB = diameter at base of live crown, D23 and D13 = diameter at lower and upper third of live crown.

The section-wise diameter distribution is displayed in Fig. 2. With the exception of one outlier, values recorded for crown diameters were less than 5 cm. There was a sharp absolute decrease in mean section diameter from crown base (DCB = 7.5 cm) to the lower section within the crown branches (D23 = 4.0 cm).

**Figure 3:**
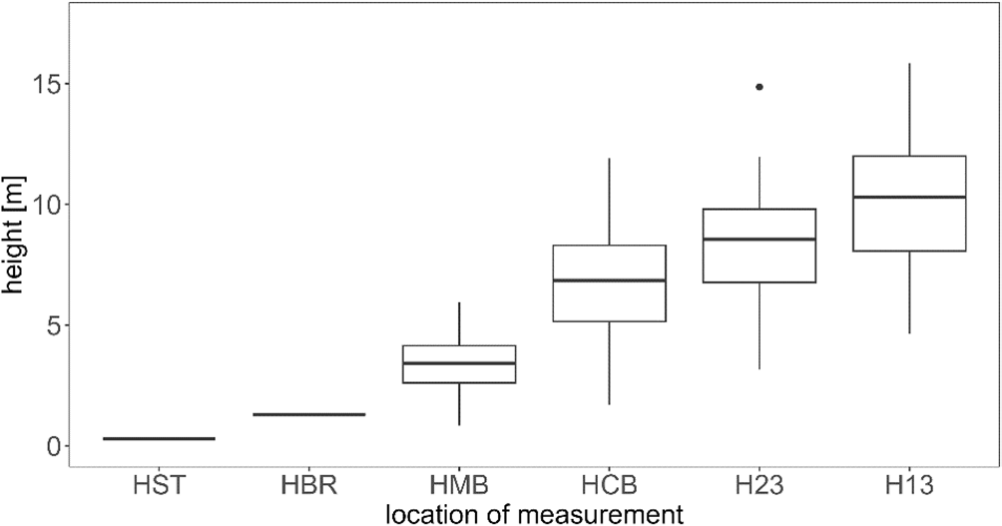
Distribution of each section height; HGR = stump height (0.3 m), HBR = breast height (1.3 m), HMB = mid-bole height, HCB = height at the base of live crown, H23 and H13 =  Height of lower and upper third of live crown.

Figure 3 shows the total tree height as well as height distribution for each section. Total tree height H ranged from 6 to 18 m. Mid-bole height HMB was recorded lower than breast height for two small trees.

### Ethical approval

The study complies with local and national guidelines and regulations.

## Results

### Fixed-effects (FE) taper models

Nineteen over-bark (o.b.) taper functions were fitted to model tree taper of black locust. Out of 19 over-bark gnls models, 15 converged and 11 had fully significant coefficient estimates. Table 2 summarizes the statistics for the trees selected for taper functions o.b. and Table 3 for the under-bark (u.b.) dataset. The model with the highest number of calibrated coefficients without non-significant terms was Eq. (11) for both o.b. and u.b. Equations 1, 9, 17 and 19 did not converge for either dataset using the gnls approach, Eq. (19) is therefore only included for the comparison in 3.2.

**Table 2:** Coefficient estimates of the converged fixed effect taper models, dependent variable: diameter over bark squared D_o.b._^2^; values in parentheses represent standard errors of the coefficients; n.s. marks non-significant coefficients at p > 0.05; values rounded to three decimals.

	β1	β2	β3	β4	β5	β6	β7	β8	β9
Equation (2) Biging^63^	1.278 (0.009)	0.359 (0.008)							
Equation (3) Bruce^61^	0.722 (0.050)	2.436 (6.907) n.s	− 152.319 (71.534)	4163.554 (2585.434) n.s	9.696 (8.391) n.s	− 222.634 (85.816)			
Equation (4) Cao^44,64^	1.18 (0.035)	− 7.087 (1.299)	9.681 (1.384)	0.369 (0.027)	0.674 (0.030)				
Equation (5) Demaerschalk^65^	1.274 (0.059)	0.906 (0.024)	0.752 (0.019)	− 0.713 (0.042)					
Equation (6) Kozak^66^	0.611 (0.050)	0.492 (0.045)							
Equation (7) Kozak^59^	0.865 (0.104)	0.836 (0.05)	0.187 (0.038)	0.311 (0.052)	− 8.285 (1.125)	− 0.804 (0.458) n.s	0.556 (0.471) n.s	0.101 (0.026)	
Equation (8) Kozak^33^	0.859 (0.038)	0.924 (0.018)	0.13 (0.032)	1.16 (0.138)	− 1.135 (0.181)	0.577 (0.078)	1.708 (0.268)	0.021 (0.017) n.s	− 0.024 (0.070) n.s
Equation (10) Lee^68^	1.471 (0.037)	0.91 (0.010)	4.953 (0.544)	− 5.193 (0.538)	2.036 (0.134)				
Equation (11) Max^40^	− 5.929 (0.758)	3.097 (0.450)	− 7.062 (1.981)	9.065 (2.063)	0.602 (0.048)	0.346 (0.040)			
Equation (12) Muhairwe^47^	19.152 (9.836) n.s	0.91 (0.013)	− 2.188 (1.514) n.s	− 2.818 (3.284) n.s	2.59 (0.499)	5.648 (2.429)	− 0.015 (0.006)	0.476 (0.143)	
Equation (13) Newberry^69^	1.021 (0.004)	0.751 (0.020)							
Equation (14) Riemer^70^	0.116 (0.047)	− 0.333 (0.166)	− 1.595 (0.319)						
Equation (15) Sharma^71^	2.121 (0.009)								
Equation (16) Sharma^72^	0.976 (0.014)	2.138 (0.017)	− 0.907 (0.187)	1.506 (0.255)					
Equation (18) Thomas^41^	− 6.429 (0.299)	1.344 (0.241)	0.015 (0.001)

**Table 3:** Coefficient estimates of the converged fixed effect taper models, dependent variable: diameter under bark squared D_u.b._^2^; values in parentheses represent standard errors of the coefficients; ns marks non-significant coefficients at p > 0.05; values rounded to three decimals.

	β1	β2	β3	β4	β5	β6	β7	β8	β9
Equation (2) Biging^63^	1.150 (0.008)	0.321 (0.007)							
Equation (3) Bruce^61^	0.586 (0.040)	1.691 (5.640) n.s	− 116.489 (58.191)	2785.956 (2069.559) n.s	9.610 (6.767) n.s	− 195.002 (69.529)			
Equation (4) Cao^44,64^	1.230 (0.031)	− 4.829 (0.386)	7.894 (1.229)	0.274 (0.016)	0.762 (0.028)				
Equation (5) Demaerschalk^65^	1.054 (0.051)	0.890 (0.025)	0.747 (0.019)	− 0.658 (0.044)					
Equation (6) Kozak^66^	0.533 (0.043)	0.460 (0.038)							
Equation (7) Kozak^59^	0.596 (0.075)	0.896 (0.046)	0.223 (0.042)	0.304 (0.048)	− 7.872 (0.929)	− 0.219 (0.378) n.s	0.664 (0.387) n.s	0.074 (0.023)	
Equation (8) Kozak^33^	0.753 (0.037)	0.914 (0.019)	0.152 (0.034)	1.057 (0.128)	− 0.732 (0.182)	0.476 (0.078)	1.118 (0.303)	0.006 (0.018) n.s	0.029 (0.080) n.s
Equation (10) Lee^68^	1.259 (0.031)	0.928 (0.01)	4.770 (0.525)	− 4.918 (0.516)	1.942 (0.128)				
Equation (11) Max^40^	− 4.970 (0.587)	2.596 (0.347)	− 5.018 (0.910)	7.671 (1.106)	0.623 (0.041)	0.302 (0.029)			
Equation (12) Muhairwe^47^	26.486 (13.288)	0.930 (0.012)	− 4.485 (1.459)	3.094 (3.085) n.s	3.064 (0.487)	0.927 (2.224) n.s	− 0.018(0.006)	0.540 (0.143)	
Equation (13) Newberry^69^	0.924 (0.004)	0.734 (0.020)							
Equation (14) Riemer^70^	0.123 (0.003)	0.110 (0.016)	0.446 (0.594) n.s						
Equation (15) Sharma^71^	2.053 (0.008)								
Equation (16) Sharma^72^	0.793 (0.011)	2.138 (0.016)	− 0.948 (0.177)	1.550 (0.243)					
Equation (18) Thomas^41^	− 5.667 (0.275)	1.326 (0.221)	0.013 (0.001)						

The subsequent ranking of the 18 models was performed for four error metrics calculated between the observed D_o.b._2 and the respective model predictions as well as AIC. Next, rankAVG was calculated as the horizontal mean of each individual rank. The top five equations by rankAVG are displayed in Table 4 (o.b. models) and Table 5 (u.b. models). Equation (8) performed best by lowest rankAVG for both the o.b. and u.b. datasets. Equation (10), Eq. (12) Eq. (7) and Eq. (11) were the next four best models. Generally, the first three models performed similarly, with only slight deviations in mean bias.

**Table 4:** Goodness-of-fit statistics for the five best performing fixed effect models (diameter over bark squared D_o.b._^2^).

	RMSE	MAE	MB	MPB	AIC	
Equation (8) Kozak^33^	17.025	9.655	1.357	− 0.067	1381	
Equation (10) Lee^68^	17.392	9.623	1.844	− 0.017	1391	
Equation (12) Muhairwe^47^	17.779	9.700	3.771	0.077	1391	
Equation (7) Kozak^59^	19.990	11.404	− 0.033	− 0.185	1418	
Equation (11) Max^40^	18.822	11.127	− 2.033	− 0.094	1435	

	rankRMSE	rankMAE	rankMB	rankMPB	rankAIC	rankAVG
Equation (8) Kozak^33^	1.000	1.010	1.360	1.210	1.000	1.116
Equation (10) Lee^68^	1.080	1.000	1.490	1.000	1.330	1.180
Equation (12) Muhairwe^47^	1.160	1.030	2.010	1.250	1.330	1.356
Equation (7) Kozak^59^	1.620	1.580	1.000	1.710	2.230	1.628
Equation (11) Max^40^	1.370	1.490	1.540	1.330	2.800	1.706

RMSE = Root Mean Square Error, MAE = Mean Absolute Error, MB = Mean Bias, MPB = Mean Percent Bias, AIC = Akaike’s Information Criterion.

**Table 5:** Goodness-of-fit statistics for the five best fixed effect models (diameter under bark squared D_u.b._^2^).

	RMSE	MAE	MB	MPB	AIC	
Equation (8) Kozak^33^	14.925	7.909	0.920	− 0.132	1307	
Equation (10) Lee^68^	15.236	8.022	2.024	− 0.006	1314	
Equation (12) Muhairwe^47^	15.292	7.968	2.874	0.018	1312	
Equation (7) Kozak^59^	16.812	9.202	− 0.072	− 0.168	1342	
Equation (11) Max^40^	16.207	9.137	− 2.534	− 0.133	1346	

	rankRMSE	rankMAE	rankMB	rankMPB	rankAIC	rankAVG
Equation (8) Kozak^33^	1.000	1.000	1.280	1.520	1.000	1.160
Equation (10) Lee^68^	1.030	1.030	1.640	1.000	1.230	1.186
Equation (12) Muhairwe^47^	1.030	1.020	1.910	1.050	1.160	1.234
Equation (7) Kozak^59^	1.160	1.330	1.010	1.660	2.140	1.460
Equation (11) Max^40^	1.110	1.320	1.800	1.520	2.270	1.604

RMSE = Root Mean Square Error, MAE = Mean Absolute Error, MB = Mean Bias, MPB = Mean Percent Bias, AIC = Akaike’s Information Criterion.

Overall, the u.b. models showed lower absolute errors, which is due to these diameter values being naturally lower than their o.b. equivalent. In terms of relative errors, regarding mean percent bias (MPB), the u.b. models performed similar to the o.b. models. All models that were biased positively for o.b. were also biased with the u.b. data and vice versa.

When analyzing model performance for relative height by RMSE of D_o.b._^2^, Eq. (8) reached the lowest error in four classes (Table 6), including the lowest segment at the stump. Four other models achieved the lowest RMSE in at least a single class. As bark volume per meter decreases to the tip of the stem, the lower stem regions should be prioritized for reducing errors. It is notable that RMSE decreases from at least 25 cm for measurements in the lowest tenth to less than 5 cm in the next, while increasing again to ca. 15 cm in the third.

**Table 6:** Comparison of Root Mean Square Error (RMSE) over 10 classes of relative height (RH) for FE models of diameter over bark squared (D_o.b._^2^).

RH class	0 < RH ≤ 0.1	0.1 < RH ≤ 0.2	0.2 < RH ≤ 0.3	0.3 < RH ≤ 0.4	0.4 < RH ≤ 0.5	0.5 < RH ≤ 0.6	0.6 < RH ≤ 0.7	0.7 < RH ≤ 0.8	0.8 < RH ≤ 0.9	0.9 < RH ≤ 1	N lowest RMSE
N of meas	41	18	21	13	7	11	23	18	27	1	
Equation (4) Cao^44,64^	29.269	3.829	14.995	9.593	12.489	16.982	32.194	6.019	4.654	0.000	1
Equation (8) Kozak^33^	25.954	3.276	15.198	7.814	30.287	14.057	19.740	6.368	3.840	5.309	4
Equation (10) Lee^68^	26.217	2.887	13.249	10.961	14.564	15.181	26.129	5.814	4.320	6.020	3
Equation (11) Max^40^	28.071	3.660	15.095	10.247	10.796	16.243	29.563	5.920	4.666	5.756	1
Equation (12) Muhairwe^47^	28.583	3.854	15.671	9.757	25.861	10.068	20.064	6.424	5.770	6.347	1

As for D_u.b._^2^, Eq. (12) was the only equation to achieve the lowest RMSE in three classes (Table 7), with Eq. (8) only achieving two.

**Table 7:** Comparison of Root Mean Square Error (RMSE) over 10 classes of relative height (RH) for FE models of diameter under bark squared (D_u.b._^2^).

RH class	0 < RH ≤ 0.1	0.1 < RH ≤ 0.2	0.2 < RH ≤ 0.3	0.3 < RH ≤ 0.4	0.4 < RH ≤ 0.5	0.5 < RH ≤ 0.6	0.6 < RH ≤ 0.7	0.7 < RH ≤ 0.8	0.8 < RH ≤ 0.9	0.9 < RH ≤ 1	N lowest RMSE
N of meas	41	18	21	13	7	11	23	18	27	1	
Equation (4) Cao^44,64^	24.834	3.172	13.382	7.537	7.735	10.650	27.664	5.655	3.745	0.000	2
Equation (8) Kozak^33^	23.565	3.508	7.383	8.842	17.739	12.210	27.354	7.760	11.498	4.127	2
Equation (10) Lee^68^	24.362	2.794	8.226	7.039	21.553	11.931	18.512	5.583	3.239	3.530	2
Equation (11) Max^40^	24.872	2.604	7.699	9.980	12.197	12.844	20.917	5.079	3.642	4.343	1
Equation (12) Muhairwe^33^	26.197	3.689	8.150	7.889	21.435	9.903	16.603	5.049	3.814	4.450	3

### Comparison of over-bark fixed effect models with foreign coefficients

The first comparison between a foreign and the current Greek (GR) over-bark fixed effect model was conducted with a west-Polish model (PL) of Eq. (7)^49^. While the fitting of Eq. (7) for the Greek sample yielded two non-significant coefficients β6 and β7, the sample from Poland showed a different selection with β3, β6 and β7 being non-significant (Table 8).

**Table 8:** Coefficients for Eq. (7) calibrated by Bronisz and Zasada^49^ (fixed effects, over bark).

Coefficient	Estimate	Coefficient	Estimate
β1	0.962	β5	– 7.643
β2	0.983	β6	n.s
β3	n.s	β7	n.s
β4	0.289	β8	0.061

Secondly, Eq. (19) was fitted in the North-Eastern United States^60^. In their original study, FE coefficients for D_o.b._2 were provided for a sample of “species group 18” trees (Table 9). No coefficient was reported as non-significant. However, in the present study, the Greek sample produced non-significant estimates for β1, β2 and β10 using the o.b. dataset. Furthermore, the addition of both random effects ${\varphi }_{{1}_{j}}$ and ${\varphi }_{{2}_{j}}$, as they are included in the equation of the original study, did not lead to a converging model. The Greek model fit is therefore a FE model rather than a mixed model.

**Table 9:** Coefficients for Eq. (19) calibrated by Westfall and Scott^60^ (fixed effects, over bark diameter squared).

Coefficient	Estimate	SE	Coefficient	Estimate	SE
β1	1.298	0.06	β6	0.456	0.03
β2	0.768	0.05	β7	9.051	1.10
β3	0.541	0.02	β8	0.024	0.00
β4	4.182	0.77	β9	1.668	0.20
β5	0.068	0.01	β10	0.177	0.01

**Figure 4:**
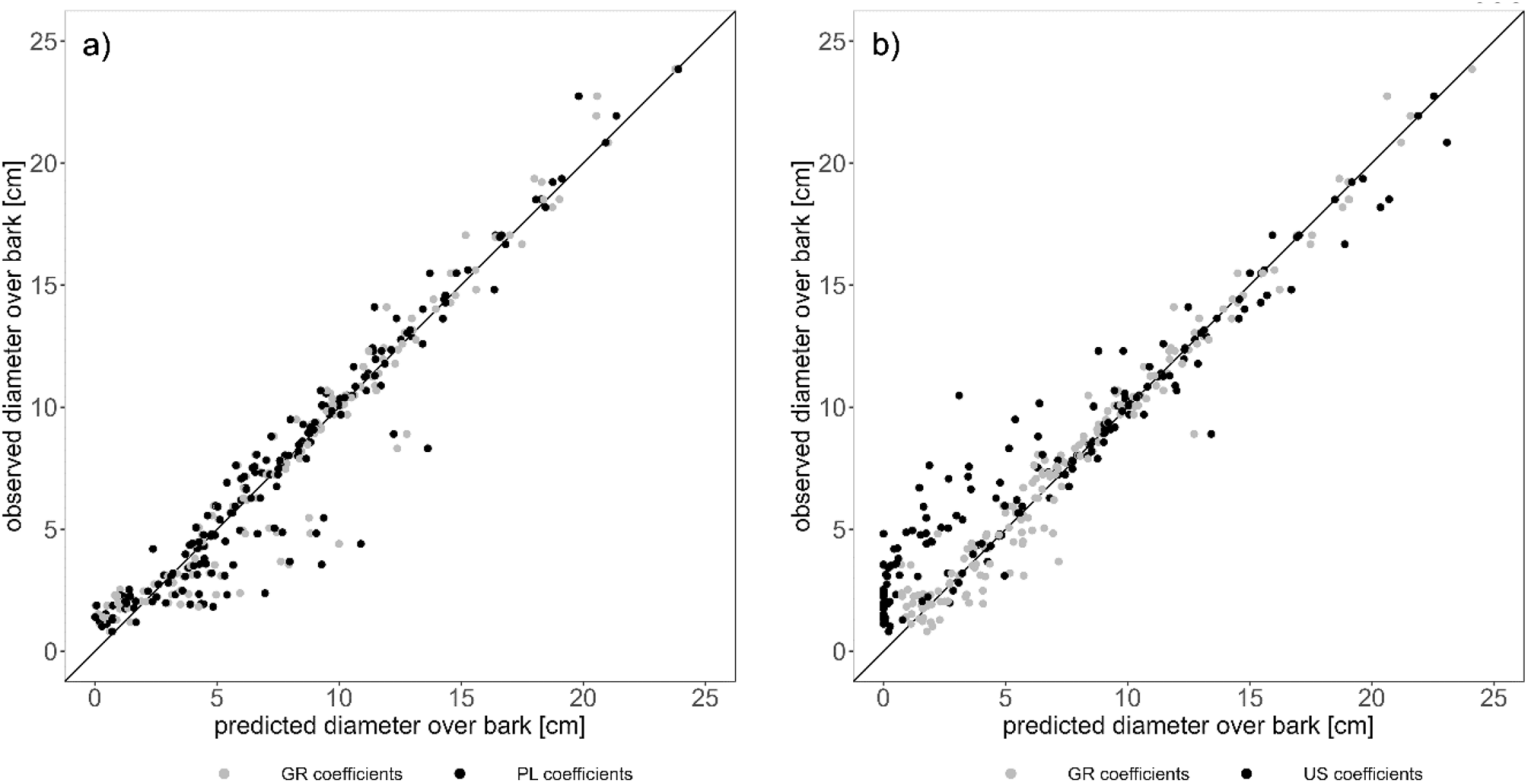
Actual section diameter (D o.b.) vs. GR and PL predictions of Eq. (7) (**a**) and vs. GR and US predictions of Eq. (19) (**b**).

Figure 4a shows the comparison of actual section diameter with both locally fitted coefficients (GR) and coefficients from the Polish model (PL). RMSE was calculated between the GR model predictions and the observed D, as well as with the foreign model’s predictions. While a visual inspection only showed slight differences in deviation from the equality line for PL points, RMSE was about 13% higher compared to the GR model fit (GR RMSE = 1.234 cm vs PL RMSE = 1.387 cm).

Observing the values predicted by the North-Eastern US model coefficients however, errors were much higher than for the Greek model (Fig. 4b). The increase in RMSE amounted to 218% over the Greek *nlsLM()* function for fitting nonlinear least squares models equivalent (GR RMSE = 0.912 cm and US RMSE = 1.991 cm).

### Mixed-effect (ME) taper models

Kozak’s 2004 Eq. (8)^33^ proved to be the best performing FE model for both the o.b. and u.b. dataset. In the subsequent grid search for the best allocation of 1, 2 or 3 random effects, the o.b. model assigned with random effects for parameters β4 and β8 reached the best goodness-of-fit out of 70 created models and β6 and β8 for u.b. (Table 10). There was a substantial increase in model accuracy by all four means of error calculation, while AIC also decreased by 31 for o.b. and 35 for u.b.

**Table 10:** Goodness-of-fit comparison for the Eq. (8) fixed-effect (FE) model and best mixed-effect (ME) model.

Over bark	RMSE	MAE	MB	MPB	AIC
FE	17.025	9.655	1.357	− 0.067	1381
ME	14.594	8.580	0.095	− 0.111	1350

Under bark	RMSE	MAE	MB	MPB	AIC
FE	14.925	7.909	0.920	− 0.132	1307
ME	10.260	5.828	− 0.108	− 0.075	1272

RMSE = Root mean square error, MAE = Mean Absolute Error, MB = Mean Bias, MPB = Mean percent bias, AIC = Akaike’s information criterion.

While the FE model featured the two non-significant coefficients β8 and β9 in both o.b. and u.b. calibrations, the o.b. and u.b. ME model also showed this behavior with the same two non-significant coefficients (Table 11). The fixed coefficients of both ME models are displayed in Table 11. Both random effects β4j and β8j of the o.b. ME model showed a standard deviation of less than 0.02. The correlation between the two effects reached − 1.

**Table 11:** Calibrated fixed effect coefficients for the best ME models of the over and under-bark dataset.

Over bark		Under bark	
β1	0.911	β1	0.768
β2	0.966	β2	0.941
β3	0.068	β3	0.118
β4	1.029	β4	1.274
β5	− 1.225	β5	− 1.039
β6	0.618	β6	0.496
β7	1.910	β7	1.530
β8	0.031 n.s	β8	0.027 n.s
β9	− 0.069 n.s	β9	0.029 n.s
Variance power estimate δ mean ± standard deviation	3.361 ± 0.554		4.801 ± 0.839
Correlation structure order 1	− 0.334		− 0.209
Random effects structure			
β4j SD	0.018	β6j SD	0.100
β8j SD	0.019	β8j SD	0.048
β4_j_-β8_j_ correlation	− 1.000	β6_j_-β8_j_ correlation	− 1.000
Likelihood ratio test			
fixed effects AIC	1381.192	fixed effects AIC	1306.552
mixed effects AIC	1349.640	mixed effects AIC	1272.148
Likelihood ratio	37.552	Likelihood ratio	40.405
p-value	3.516*10^–08^	p-value	8.745*10^–09^

Random effects structure of the best ME model; likelihood ratio test results comparing with the respective fixed effect model; Estimate of the power function to model the residual variance as mean ± standard deviation; Estimate of the first-order autoregressive structure;

The likelihood ratio test between the FE and ME models of Eq. (8) revealed a statistically significant difference for o.b. as well as for the u.b. models.

**Figure 5:**
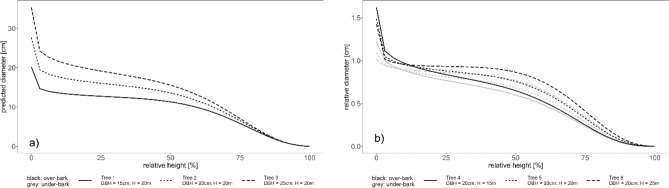
Sample taper curves for over and under-bark of relative diameter along relative height for the fitted Eq. 8. mixed effects model. (**a**) Varying diameter at breast height DBH = 15, 20, 25 cm and fixed tree height H 20 m. (**b**) Varying H = 15, 20, 25 m and fixed DBH = 20 cm.

The over- and under-bark taper curves from the Eq. (8) mixed model predictions for three sample combinations of DBH, 15 cm, 20 cm and 25 cm at the same tree height H = 20 m displayed the largest differences at the center of the stem (Fig. 5 a). A similar behavior was observed for the taper curves at different tree heights of 15, 20 and 25 m but with a fixed DBH = 20 cm (Fig. 5b).

### Stem volume

**Figure 6:**
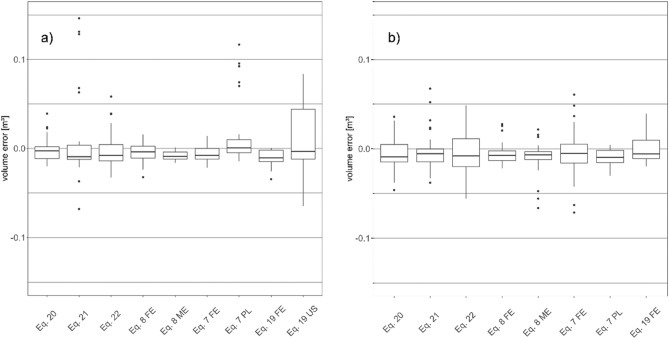
Stem volume error distribution [m^3^] for three volume models and integrated Eq. 7, Eq. 8 compared to reference volume; left: over bark– right: under bark.

All three volume equations showed a tighter volume error spread compared to the numerically integrated FE models in the o.b. category (Fig. 6). In contrast, the taper models were less biased with medians closer to zero. Overall, stem volume errors ranged between − 0.07 and 0.15 m^3^ o.b. and for u.b. from − 0.07 to 0.07 m^3^. The Eq. (8) ME model surpassed all other equations for o.b. but not for u.b. where the FE model had fewer outliers and less bias. Calculating o.b. volume using the PL coefficients, a slightly wider spread was observed compared to the respective Greek equivalent. For the US coefficients, the errors were also higher compared to the Eq. (19) FE model. However, there were no u.b. coefficients available for the said equation.

The Kruskal–Wallis test indicated statistically significant differences in volume error depending on the equation used for prediction for the o.b. dataset, but not the u.b. errors. A subsequent Dunn-test revealed differences between the Eq. 8 ME model with only the Eq. (7) PL and Eq. (19) US errors. The volume errors from the FE models and volume equations were not found to significantly differ among each other.

## Discussion

To model both o.b. and u.b. taper of black locust, 18 different equations were analyzed. The models were ranked using a relative ranking of five separate goodness-of-fit metrics: root mean square error (RMSE), mean absolute error (MAE), mean bias (MB), mean percentage bias (MPB) and Akaike’s Information Criterion (AIC). The variable exponent equation Eq. (8)^33^ ranked best for both o.b. and u.b. datasets. Kozak’s^59^ variable exponent model Eq. (7), which had been identified as the best performing model for black locust in Bronisz and Zasada^49^, was found to be the fourth-best model in this study. In their own study, Kozak^33^ found Eq. 8 to be superior to Eq. (7) for 38 species groups in British Columbia, Canada. Rojo et al.^83^ found Eq. (8) to be the best equation among 31 taper functions applied to Maritime pine (*Pinus pinaster* Ait.). Poudel et al.^84^ also observed that Eq. (8) performed best for Douglas fir (*Pseudotsuga menziesii* Franco) compared to three variable-exponent equations.

Variable exponent equations of Eq. (12) (Muhairwe)^47^ and Eq. (10) (Lee et al.)^68^ were the second and third best models respectively, adding to the hypothesis that complex, i.e. non-parameter-parsimonious equations perform better than their less complex counterparts. This has been observed in multiple studies comparing several taper equations, including de-Miguel et al.^48^ and Bronisz and Zasada^49^. These equations aim to achieve good predictive ability for all parts of the stem, which was confirmed by measuring RMSE by 10 classes of relative height. Kozak’s Eq. (8)^33^ scored best four times for o.b. and twice for u.b., followed by further variable-exponent models.

Recently published taper models for black locust from Poland^49^ with Eq. (7), Kozak’s^59^ variable exponent model, and Eq. (19), the Westfall and Scott 2010 model^60^, achieved error values of 13 and 218% of the Greek model fit. Therefore, neither taper model calibrated for foreign datasets proved suitable for predicting stem diameters of the present sample. As these two studies are among, if not the only, applications of taper modelling for black locust, further research is needed to provide adequate predictions for stands within Europe and North America. In addition, the tapering properties of planted black locust in post-mining areas might differ from black locust growing in forest sites due to different growing conditions on disturbed soils. Research showed that even among and within black locust families there is a large amount of genetic variation for growth, form, thorn length and other important traits^85^.

An exhaustive search for all possible combinations of up to three random effects was performed on the basis of the best FE taper equation. Thus, the addition of two random effects to the generalized least-squares model of Eq. (8) provided a substantial decrease in errors for both o.b and u.b. datasets, as the random effects allowed for slight adjustment to the individual growth characteristics of each tree. Variance modelling, i.e., applying weights to the FE and ME models on a single-tree basis depending on a power-type function of DBH was included to account for heteroscedasticity.

In the direct comparison between the three stem volume functions and numerically integrated taper equations, the ME model was able to surpass the other methods slightly. However, as all three volume equations presented a negative bias, predictions could be adjusted. That said, given the small sample size of 30 stems in this study, bias should be investigated on a larger sample that may also expand the diameter and height range of these relatively young trees. Without destructively sampled allometric data and a locally calibrated model, the three volume equations from previous studies^74–76^ serve as a rather precise means of estimating o.b. and u.b. stem volume for the Greek plantation black locust trees. Therefore, diameter at breast height and total tree height would be the only required measurements of the respective sample trees. Under-bark diameter would then need to be sampled via a bark gauge along the stem to verify an additional model for stem wood volume.

Valentine and Gregoire^86^ point out that the butt part of the stem is the most error-prone in taper modelling. In contrast, the number of measurements taken in this area is relatively low, with only stump height and breast height in most taper datasets. Westfall and Scott^60^ also indicate that using few measurements in the lowest part of the stem might result in an underestimation of errors. This issue is present with this study especially, as the small dataset of just 180 measurements may only serve as a minimum sample. In turn, the primarily desired area of application in the Western Greece Lignite Center covers about 2500 ha. However, Yang and Burkhart^87^ found that withholding certain classes of DBH for parametric fitting approaches, i.e. the smallest or largest diameter trees, did not effectively influence the predictive ability of taper functions Eq. (8) and Eq. (11) for the rest of their sample. Therefore, the present dataset of young trees used for calibration might be sufficient to predict larger tree diameters in the growing stands of the Western Greece Lignite Center. Suitable trees should be measured in 10 years to confirm the taper models applied for the 2020/2021 sample.

Nevertheless, with more sample trees from Greece and the broader Balkan region, a larger dataset could be obtained. With plans to phase out the use of fossil resources (especially lignite) for energy in Europe in line with international agreements to limit the effects of human-induced climate change, most open-cast mines will be depleted or abandoned within coming decades. Exotic species can be recommended for the rehabilitation of former open cast bare coal mine spoils due to their fast growth and establishment^88^. Black locust is an appropriate tree species for the regeneration of post-mining sites. However, the growth and taper of black locust on degraded sites are not well studied. Some studies have focussed on black locust as a suitable tree species for agroforestry systems^14,85,89–92^. In short-rotation agroforestry systems, the harvested annual yield is between 8.1 and 9.7 t_odt_ ha^−1^ year^−1^^14,90^. In such systems, the aboveground biomass is mostly used to produce bioenergy.

The cited research from several European countries, such as Germany, Greece and Ukraine, shows that black locust is indeed a suitable species for reforesting these disturbed sites. The production of woody biomass in unfavorable forest vegetation conditions of disturbed lands in combination with effective soil erosion protection is increasingly important to respond to the growing demand for bioenergy and conservation^2,17,21,93,94^. Due to its environment-creating and productive functions, black locust is a very promising tree species in the fight against desertification and climate change as global challenges. However, as a non-native and invasive species, it should be used for land reclamation purposes in a clearly defined area of contaminated sites and for the production of durable and long-lived wood products if it is to be regarded as environmentally justified^18,21^. A wider network of allometric datasets from reforestation areas, including taper and volume models, would be beneficial for more precise predictions of timber assortments, volume and carbon accounting for post-mining landscapes in the near future.

## Conclusions

The following conclusions can be drawn from the present study at the black locust restoration plantations of the Lignite Centre of Northwestern Greece:

- From the eighteen different taper equations that were ranked, Kozak’s^33^ model performed best for both the over- and under-bark data followed by the Muhairwe’s^47^, Lee’s et al.^68^, Kozak’s^59^ and Max and Burkhart’s^40^ equations.
- ME model regression substantially increased the over and under-bark taper model accuracy by all four means of error calculation, AIC also decreased by over 30 units and presented the largest decrease in residual variance for both responses $D^{2}$ and $RH$ over the original FE models.
- A comparison for weighted fixed effects o.b. showed that Kozak’s 1995 variable exponent model^59^ exhibited two non-significant coefficients in contrast to three other non-significant coefficients in Bronisz and Zasada’sPolish dataset^49^.
- Stem volume equations proved to be performing well and close to the numerically integrated taper equations.

## Data Availability

The experimental data that support the findings of this study are available in the BonaRes repository site with the identifier: Wilms, F., Berendt, F., & Spyroglou, G. (2024). Taper dataset for planted Black locust (Robinia pseudoacacia, L.) in Greek post-mining areas [Data set]. Leibniz Centre for Agricultural Landscape Research (ZALF). 10.4228/ZALF-KH4C-0B85.
